# Central-line associated bloodstream infections in a tertiary care children’s University hospital: a prospective study

**DOI:** 10.1186/s12879-016-2061-6

**Published:** 2016-12-01

**Authors:** Elisabetta Venturini, Carlotta Montagnani, Alessandra Benni, Sabrina Becciani, Klaus Peter Biermann, Salvatore De Masi, Elena Chiappini, Maurizio de Martino, Luisa Galli, on behalf of the CLABSI (central-line associated bloodstream infections) study group, M. Alterini, M. Anziati, C. Baccini, T. Bardelli, M. Baroni, F. Catarzi, E. Ciriello, S. Cozza, G. Diani, L. Ficozzi, S. Francini, P. Gervaso, L. Guidozzi, A. Ingargiola, L. Lega, L. Lenzi, A. Lodi, S. Loru, M. Morganti, T. Morrione, C. Neri, F. Paoli, E. Pierucci, M. Prato, R. Putrino, S. Sollai, V. Tintori, A. Tondo, D. Trefoloni

**Affiliations:** 1 Department of Health Sciences, University of Florence, Meyer Children’s University Hospital, Florence, Italy; 2 Meyer Children’s Hospital Healthcare Associated Infection Control Committee, Florence, Italy; 3 Pediatric Infectious Diseases Division, Department of Pediatric Medicine, Meyer Children’s University Hospital, viale Pieraccini 24, I-50139 Florence, Italy

**Keywords:** CLABSI, Children, Central-line, Infection

## Abstract

**Background:**

The central-line associated bloodstream infections (CLABSI) are the most common healthcare-associated infections in childhood. Despite the international data available on healthcare-associated infections in selected groups of patients, there is a lack of large and good quality studies. The present survey is the first prospective study monitoring for 6 months the occurrence of central-line associated bloodstream infections in all departments of an Italian tertiary care children’s university hospital.

**Methods:**

The study involved all children aged less than 18 years admitted to Meyer Children’s University Hospital, Florence, Italy who had a central line access between the October 15^th^, 2014 and the April 14^th^, 2015. CLABSI were defined according to the Center for Disease Control and Prevention criteria. CLABSI incidence rates with 95% confidence limits were calculated and stratified for the study variables. For each factor the relative risk and 95% confidence intervals were evaluated. Statistical analysis was performed using the statistical software SPSS for Windows, version 22.0 (SPSS Inc., Chicago, IL), *p* < 0.05 was considered statistically significant.

**Results:**

CLABSI rate was 3.73/1000 (95% CI: 2.54–5.28) central line-days. A higher CLABSI incidence was seen with female gender (*p* = 0.045) and underlying medical conditions (excepting prematurity, surgical diseases and malignancy) (*p* = 0.06). In our study 5 infections, were caused by extended-spectrum β-lactamase producing organisms and in one case by carbapenem-resistant *Klebsiella pneumoniae*.

**Conclusions:**

Our study confirms the spreading of multi-resistant pathogens as causes of healthcare associated infections in children. An increased incidence rate of CLABSI in our study was related to underlying medical conditions. Pediatric studies focusing on healthcare infections in this type of patients should be done in order to deepen our understanding on associated risk factors and possible intervention areas.

## Background

The central line associated bloodstream infections (CLABSI) are the most common healthcare-associated infections (HAIs) in childhood. The results of the *International Nosocomial Infection Control Consortium* surveillance study from January 2007 to December 2012 in pediatric intensive care units (PICU) in Latin America, Asia, Africa and Europe, showed CLABSI rate of 6.1/1000 (95% CI: 5.7–6.5) central line-days [1]. The 2014 report of the *European Centre for Disease Prevention and Control* (ECDC) estimated a mean device-adjusted rate in patients staying in an ICU for more than two days was 3.0 CLABSI episodes per 1000 central line-days (IQR: 0.5–4.1) [2]. A cohort study performed from 2007 to 2012 in 173 neonatal intensive care unit (NICU) and 64 PICU reporting data to the *Centers for Disease Control and Prevention’s National Healthcare Safety Network* in United States, showed a reduction of CLABSI rates from 4.9 to 1.5/1000 central line-days in NICU and from 4.7 to 1.0/1000 central line-days in PICU in 5 years time [3]. A trend of infection rate reduction was seen also in a prospective 2-year quality improvement British project conducted in 21 PICU with overall 34,635 central line-days analyzed [4].

Despite the international data available on HAIs in selected groups of patients, there is a lack of large and good quality studies. The best methodology to obtain reliable data would be a continuous prospective study. However, this type of study would require dedicated staff and enormous financial resources. Short prospective study or Point-prevalence survey are possible strategies. In the first case, data are collected about a certain period of time, giving a good estimate of the global burden and being cost-saving. The point-prevalence survey gives a punctiform picture (usually a day), allows collecting data in an easy and low-cost way but it is less accurate than the previous method. A Canadian point-prevalence survey was done on about 1353 pediatric patients with central-line access or with assisted-ventilation. Data were collected for 24 h in 30 hospitals. One hundred-eighteen patients (8.7%) had a HAI, of those 38 events (30.7%) were CLABSI [5]. A prospective study performed in 29 NICU in United States between October 2006 and December 2007 found that the risk of CLABSI is very low during the first week of catheterization and especially with lines inserted in the jugular vein, whereas it is increased in oncological and gastrointestinal patients and in case of prolonged catheterization [6]. Further recent studies estimated that CLABSI results in additional hospital costs on a per-case basis of 45,000 dollars [7–9]. The available studies are usually limited to selected groups of patients as oncological children or patients admitted to PICU. There are few prospective studies performed in a whole hospital in order to investigate the global incidence of HAIs. A Swiss study performed in a single institution between April 2008 and March 2009 on 152 patients showed an overall CLABSI incidence of 0.95/1000 central line-days. In this study, CLABSI incidence varied by type of catheter and by patient’s age, with the highest risk in neonates with Silastic® percutaneous central line [10].

Available data regarding children’s HAIs in Italy are limited to selected group of patients. A study done in Naples on 120 oncologic children found a positivity of blood cultures from central line in about one third (28.3%; 128/425 samples) of cases clinically suspected for infection [11]. There are two prospective studies performed in Turin: the first included 748 oncologic or immunologic patients within 7 years, registering 174 episodes of CLABSI [12], whereas the second enrolled 153 children with previous cardio-surgery, founding a CLABSI incidence of 11.7/1000 central line-days [13]. The only point-prevalence survey available in the Italian pediatric population was set in a large tertiary care children’s hospital in Rome between 2007 and 2010. A two-weeks period monitoring was performed each year of the study, with 1506 patients included. Overall, 102 (6.8%) HAIs were identified, one third of which were CLABSI [14].

The present study is the first prospective study monitoring for 6 months the occurrence of CLABSI in all departments of a tertiary care children’s University hospital.

## Methods

### Study setting

The Meyer Children’s University Hospital is a 198-bed tertiary care university hospital in Florence, Italy. It is the Tuscany Region Pediatric Hospital, but one fourth of children come from other Italian Regions or from abroad. The hospital is structured for intensity of care, with two intensive care units (NICU and PICU), and Departments of oncology, surgery, neuroscience unit (including also neurosurgery patients) and a bone marrow transplant unit. Moreover, there are two medical wards for 14 pediatric medicine sub-specialties, and an unit for elective specialty admissions. The annual hospital inpatient admissions were 8898 in 2014, for overall 53,988 admission-days.

### Study population

The study involved prospectively children aged less than 18 years admitted to Meyer Children’s University Hospital who had a central line access inserted between the October 15^th^ 2014 and April 14^th^ 2015.

### Data collection

During the study period the global number of children admitted to the hospital was monitored daily and the patients meeting the inclusion criteria were enrolled in the study. The data were collected daily by the trained healthcare staff involved in hospital infection control. Ethical approval was not required because the survey was carried out for surveillance purposes according to the criteria of hospital good clinical practice. The surveillance of CLABSI was performed in all the intensive care, medical, surgical and neonatal wards. The data were collected using a standardized format. The data were collected anonymously and entered in the study database, following the international guidelines for data protection.

For each child the following data were entered into the study database: demographic data, reason for admission, underlying illness, type of device, antibiotic treatment. Reasons to stop the follow-up were: hospital discharge, device removal or infection event related to the device. Clinical, hematological and microbiological criteria for diagnosis were recorded in case of infection. The pathogen identified was also documented.

### Case definitions

CLABSI were defined according to the Center for Disease, Control and Prevention (CDC) criteria [15]. CLABSI are defined as laboratory-confirmed bloodstream infection where central line or umbilical catheter was in place for >2 calendar days on the date of event and the line was in place on the date of event or the day before [15]. In order to capture data only about infections acquired during the hospitalization, the definition of CLABSI was considered valid only if the positive blood culture and clinical signs/symptoms of infection occurred after at least 48 h from admission.

### Statistical analysis

Median and interquartile range (IQR) was calculated for continuous measurement in the study groups (i.e., age). Categorical data were compared using the Chi-squared test (or Fisher’s exact test, when expected cell sizes were smaller than five). CLABSI incidence rates with 95% confidence limits were calculated and stratified for the study variables. Specifically, the variables included in the analysis were: age, gender, underlying illness, type of device, treatment. For each factor the relative risk (RR) and 95% confidence intervals (95% CI) were evaluated. Statistical analysis was performed using the statistical software SPSS for Windows, version 22.0 (SPSS Inc., Chicago, IL), *p* < 0.05 was considered statistically significant.

## Results

Overall, 388 children with central line access were enrolled during their hospital stay, resulting in 7783 catheter observation-days. The median age was 21 months (IQR: 3–105.5). The underlying conditions of the children enrolled in the study were: medical (*n* = 135, 34.8%), oncological (*n* = 118, 30.4%), surgical (*n* = 116, 29.9%) and prematurity (*n* = 19, 4.9%). Median catheter dwell time during hospital admission was 4 days (IQR: 2–9).

During the study period there were 29 episodes of CLABSI in 26 patients. In particular, two children had respectively 2 and 3 infections during the observation period. The characteristics of the children with CLABSI are reported in Table 1.

**Table 1:** Characteristics of the 26 children with CLABSI during the study period

Age (months), median [IQR]	11.5 [4–37]
Gender, n (%)	
- Male	10 (38.5)
- Female	16 (61.5)
Underlying disease, n (%)	
- Medical	15 (57.7)
- Oncological	6 (23.1)
- Surgical	4 (15.4)
- Neonatological	1 (3.8)
Catheter type, n (%)^a^	
- Port-a-Cath	2 (6.9)
- PICC	3 (10.4)
- Short term	11 (37.9)
- Broviac	13 (44.8)
Catheter days, median [IQR]	13 [4–20]
Ward, n (%)^a^	
- ICU	9 (31)
- NICU	4 (13.8)
- Medical wards	9 (31)
- Oncology	4 (13.8)
- Neurology and Neurosurgery	1 (3.5)
- Surgery	2 (6.9)
Treatment, n (%)^a^	
- Yes	24 (82.8)
- No	2 (6.9)
- Unknown	3 (10.3)

*IQR* interquartile range, *PICC* peripherally inserted central catheters, *ICU* intensive care unit, *NICU* neonatal intensive care unit

^a^calculated on the total number of CLABSI event (*n* = 29)

The global incidence of CLABSI was 3.73/1000 (95% CI: 2.54–5.28) central line-days.

The risk of infection in our study population was significantly correlated to female gender (RR = 2.17, 95% CI: 1.02–4.86, *p* = 0.045). Considering the catheter type, the incidence of CLABSI appeared higher for Broviac and short-term catheters, resulting almost double than Port-a-Cath and peripherally inserted central catheters (PICC). This difference could be explained because in our setting PICC are used mostly for antibiotic treatment compared to Broviac catheters which are mainly used for parenteral nutrition and in oncology patients.

The highest rate of CLABSI resulted in children with an underlying medical condition (i.e., respiratory, renal or cardiac diseases, neurological or metabolic conditions). In this group of patients, the number of infections were 16 for 2951 central line-days, with an incidence rate of 5.42/1000 catheter-days (IQR 3.10–8.80) and a RR of 2.01 (95% CI: 0.96–4.28), *p* = 0.06. The risk of CLABSI was significantly higher in patients within the respiratory sub-group compared to all the other conditions (RR = 4.38, 95% CI: 1.30–11.74, *p* = 0.021). Patients admitted to intensive care unit and to medical wards had a higher rate of infection (respectively 5.42 and 5.69/1000 catheter-days) compared with the other units; however, this result was not statistically significant. The incidence of CLABSI by population characteristics is reported in Table 2.

**Table 2:** Distribution of CLABSI incidence by population characteristics

Population characteristics	Catheter days (n)	CLABSI (n)	Incidence of CLABSI (n/1000 catheter days)	95% CI
Age:				
- ≤ 1 year	3196	14	4.38	2.39, 7.35
- >1 year	4587	15	3.27	1.83, 5.39
Gender:				
- Male	4149	10	2.41	1.15, 4.43
- Female	3634	19	5.23	3.15, 8.16
Underlying disease:				
- Medical	2951	16	5.42	3.10, 8.80
- Oncological	2923	8	2.74	1.18, 5.39
- Surgical	1577	4	2.54	0.68, 6.49
- Neonatological	332	1	3.01	0.04, 16.76
Catheter type:				
- Port-a-Cath	775	2	2.58	0.29, 9.32
- PICC	1269	3	2.36	0.47, 6.90
- Short term catheter	2432	11	4.52	2.25, 8.09
- Broviac	2959	13	4.39	2.34, 7.51
- Umbilical vein catheter	105	0	–	–
- Unknown	243	0	–	–
Ward:				
- ICU	1660	9	5.42	2.47, 10.29
- NICU	1377	4	2.90	0.78, 7.44
- Medical ward	1581	9	5.69	2.60, 10.81
- Oncology and BMT	2015	4	1.98	0.53, 5.08
- Neurology and Neurosurgery	449	1	2.23	0.03, 12.39
- Surgery	587	2	3.41	0.38, 12.30
- Speciality ward	114	0	–	–

*CI* confidence interval, *CLABSI* central-line associated bloodstream infections, *PICC* peripherally inserted central catheter, *ICU* intensive care unit, *NICU* neonatal intensive care unit, *BMT* bone marrow transplant

**Fig. 1:**
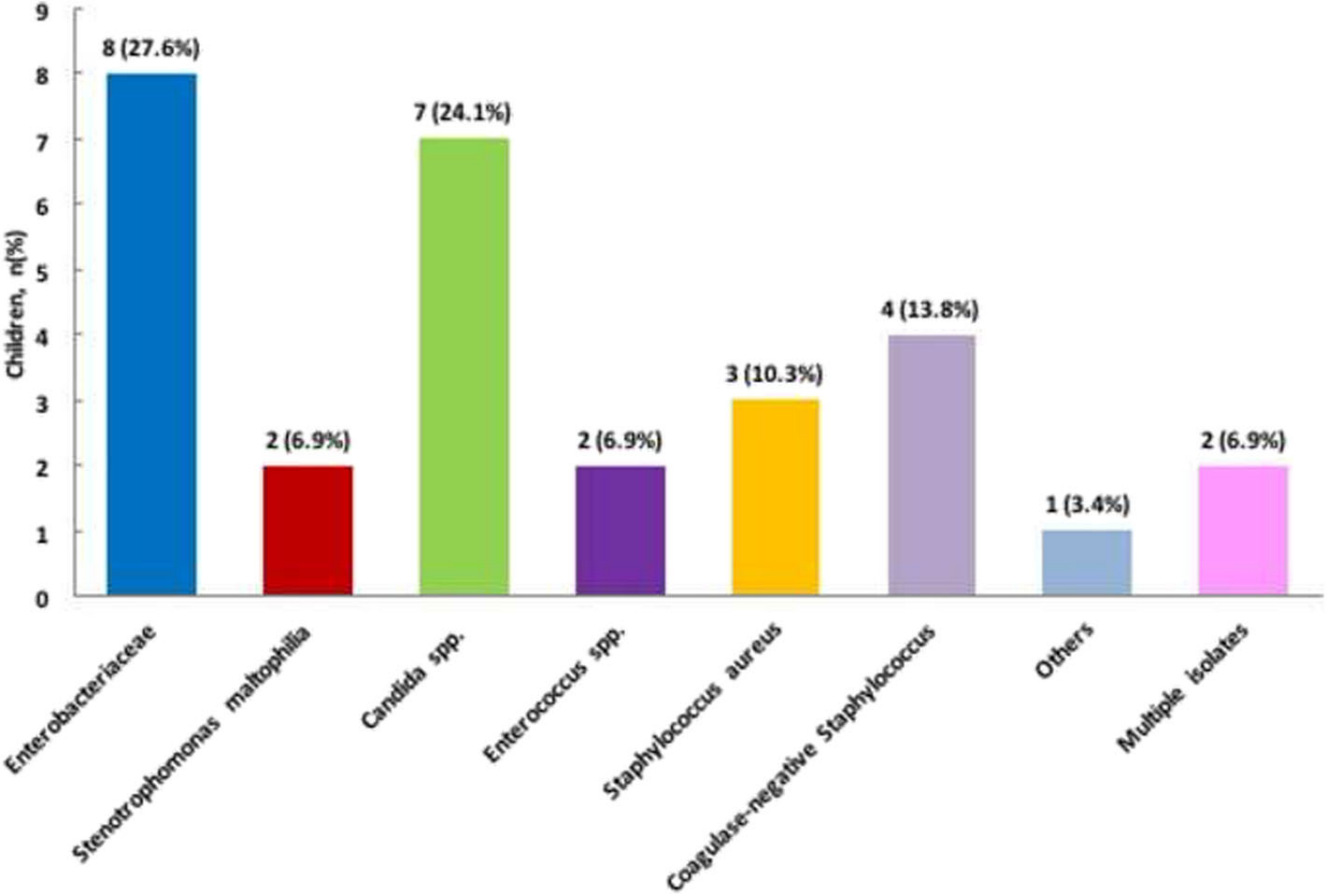
Microorganisms identified in children with CLABSI. The complete list of the microorganisms found is displayed in the figure. The pathogens isolated in patients with CLABSI are mainly *Enterobacteriaceae* and *Candida spp*. Five infections, out of the 9 cases caused by *Enterobactriaceae* identified are due to extended-spectrum β-lactamase producing organisms (3 *Escherichia coli*, 2 *Klebsiella pneumonia*e) and in one case by carbapenem-resistant *Klebsiella pneumoniae*. Specifically, *Candida albicans* has been identified in 5 (71.4%) patients and *Candida parapsilosis* in other 2 (28.6%) children

In more than half of the patients the pathogens identified were *Enterobacteriaceae* and *Candida spp*. The complete list of the microorganisms found is displayed in Fig. 1.

## Discussion

The present study is designed to assess the incidence rate of CLABSI in a whole pediatric children tertiary care hospital in a 6-months prospective surveillance. In our study CLABSI rate is 3.73/1000 (95% CI: 2.54–5.28) central line-days. Comparing studies on CLABSI is challenging due to different study population and methodology used. Only another prospective study, conducted in a whole pediatric hospital, is available in literature [10]. This study included 152 patients for overall 14,752 catheter observation days, with a CLABSI rate of 0.95/1000 central line-days [10]. This lower CLABSI rate should be interpreted considering that, differently from our survey, Wagner and colleagues enrolled mainly neonatal, hematological/oncological and surgical patients. If we consider only these types of patients in our study, the CLABSI rate decreases to 2.69/1000 catheters-days (95% CI: 1.43–4.60). Moreover, the authors concluded that the heterogeneous case mix of the patients enrolled, including also out-patients settings, accounts for the low CLABSI rate found [10]. On the contrary, our study is conducted on inpatients only; therefore, the case mix index of our study population could be estimated on the basis of the average diagnosis-related group (DRG) weight, which was 1.48 in 2014.

Moreover, the present study can be compared with two previous one-month prospective surveys carried out in 2012 and in 2013 in our tertiary-care children’s university hospital [16]. The data collected were respectively on 97 children with CLABSI (935 central line-days) in 2012 and on 122 children (1123 central line-days) in 2013. CLABSI incidence rate found in the two reports was respectively 3.21/1000 catheter days (95% CI: 1.03–9.95) in 2012 and 1.78 (95% CI: 0.45–7.12) in 2013 [16]. CLABSI incidence rate find in the present study almost overlaps with the one found in 2012 survey, but it results higher than the 2013 ones. This fact is probably due to the increasing case mix of our hospital, objectively documented by hospital DRG, as explained above. However, an important limitation of the previous surveys performed in our hospital was the short length of the study, which explains the wide confidence intervals obtained and may justified this difference.

In 2012, our hospital participated to the point prevalence survey coordinated by the ECDC, resulting to have a prevalence of HAIs of 8% (LC 95% 3.9–14.1), 9.1% of those were CLABSI (prevalence 1.8%, 95% CI −0,7–4.2%) [17]. On the other hand, the rate of HAIs in our hospital in 2014 was retrospectively estimated on the basis of laboratory-derived microbiological isolates, resulting 5.6/1000 hospital stay-days (300 infections during 53,988 days of hospitalization, 95% CI 4.9–6.2). Specifically, the analysis of microbiological data showed 39 positive blood cultures in children with central line access, with a CLABSI incidence of 0.51/1.000 central line-days (7605 central line- day, estimated by the prospective study), lower than the incidence of 3.4/1.000 central line-days in 2013. However, the retrospective methodology might underestimate the incidence of CLABSI.

Interestingly, our study points the attention on underlying medical conditions as an important risk factor for developing CLABSI. In the available literature this type of patients was poorly studied, as the attention focused mainly on intensive care and oncological patients [1, 3, 4, 6, 11, 12]. However, in this wide group are included children with high risk of developing catheter related infections for different reasons. For example, in gastroenterological disorders (i.e., short bowel syndrome or inflammatory bowel disease), the parenteral nutrition was seen to be an adjunctive risk for infections [10, 18]. Unfortunately, this information has not been collected in our study. Moreover, in chronic disorders as renal diseases (chronic renal failure, dialysis patients), chronic respiratory conditions (bronchodysplasia, congenital tracheobronchial anomalies) and metabolic diseases the infection risk might be increased by possible immunosuppressive mechanisms in these chronic diseases. Moreover, patients with polymalformative syndromes might require multiple devices for enteral feeding and respiratory support. These conditions could increase the risk of bacteremia and catheter colonization. The risk of infection seems to be particularly higher in the sub-group of respiratory patients. This might be related to the complexity of these patients, who might require a superior intensity of care [19–21].

The risk of developing CLABSI in our study population is almost double in patient with Broviac and short term catheters compared to Port-a-Cath and PICC. Despite few pediatric studies available on CLABSI incidence in patients with PICC [19, 21], studies directly comparing differences by catheter type are limited [10, 22]. Moreover, comparison of unrelated studies is difficult because of heterogeneity in study populations and study methods [23]. Large pediatric prospective studies focuses on identifying the device with lower infections rate are needed.

The pathogens isolated in patients with CLABSI are mainly *Enterobacteriaceae* and *Candida spp. Enterobacteriaceae* spread is an emergent cause of severe infections. These pathogens are of increasing concern due to the rise of carbapenem resistance, which has created a generation of organism resistant to multiple antibiotic classes [24]. In Italy, gram negative resistance rate to third generation cephalosporins is between 30 and 56% [25], whereas the rate of carbapenem resistant Klebsiella is around 34% in invasive infections [26]. There is a lack of prevalence data in pediatric patients.

In our study 5 infections, out of the 9 cases identified are caused by extended-spectrum β-lactamase producing organisms (3 *Escherichia coli*, 2 *Klebsiella pneumonia*e) and in one case by carbapenem-resistant *Klebsiella pneumoniae*. In our surveillance no methicillin resistant *Staphylococcus aureus* nor vancomycin resistant *Enterococcus* infections were detected. The CLABSI caused by *Candida spp.* are beyond the neonatal period thanks to the spreading use of fluconazole prophylaxis. Specifically, *Candida albicans* has been identified in 5 (71.4%) patients and *Candida parapsilosis* in other 2 (28.6%) children.

Regarding CLABSI rate, our study is designed to assess the incidence rate during patients’ hospital admission. Therefore, our results could not be extrapolated as an overall CLABSI incidence for each central line implanted, as this rate might differ if the follow-up was extended to the outpatient clinics and to the home-care. Moreover, our results might not necessarily reflect the HAIs incidence of other Italian hospitals because of different settings and case complexity. Monitoring HAIs is the best way to assess the risk factors for infection globally and specifically for each institution. Moreover, it helps in planning preventive interventions to reduce patient’s morbidity and mortality, to save costs and to control resistant-microorganisms spread. Point prevalence surveys are cheaper and easier to conduct than long prospective studies, but might underestimate the real risk. Cost-effectiveness balance should be done to choose the best monitoring strategy for each setting.

## Conclusions

Our study confirms the spreading of multi-resistant pathogens as causes of healthcare associated infections in children. An increased incidence rate of CLABSI in our study was related to underlying medical conditions. Pediatric studies focusing on healthcare infections in this type of patients should be done in order to deepen our understanding on associated risk factors and possible intervention areas.

## Abbreviations

CDC: Center for disease, control and prevention
CI: Confidence interval
CLABSI: Central-line associated bloodstream infections
DRG: Diagnosis-related group
ECDC: European centre for disease prevention and control
HAIs: Healthcare-associated infections
IQR: Interquartile range
NICU: Neonatal intensive care unit
PICC: Peripherally inserted central catheters
PICU: Paediatric intensive care unit
RR: Relative risk
